# Pump-Induced Biphasic Relaxation Model of Xe Spin in Nuclear Magnetic Resonance Gyroscopes

**DOI:** 10.3390/ma19061143

**Published:** 2026-03-15

**Authors:** Shangtao Jiang, Tengyue Wang, Xuyang Qiu, Yunkai Mao, Heng Yuan

**Affiliations:** 1 School of Instrumentation and Optoelectronics Engineering, Beihang University, Beijing 100191, China; 2 Hangzhou Innovation Institute, Beihang University, Hangzhou 310052, China; 3 Hefei National Laboratory, Hefei 230088, China; 4 Data Communication Science and Technology Research Institute, Beijing 100191, China

**Keywords:** NMRGs, spin relaxation, pump power, PBR model

## Abstract

The spin relaxation rate of Xe isotopes is a key characteristic of nuclear magnetic resonance gyroscopes (NMRGs). A pump-induced biphasic relaxation (PBR) model is proposed to describe the pump dependence of the transverse relaxation rate of ^129^Xe nuclear spin. The distribution of electron polarization is theoretically analyzed based on the Bloch–Torrey equations and the volume-averaged polarization is evaluated through NMR frequency shift measurements. Experimental results confirm the theoretical quadratic dependence between Γ and $P_{Rb}$ with a high fitting accuracy (R^2^ = 0.9969). The predicted linear (R^2^ > 0.9966) and hyperbolic (R^2^ > 0.9942) regimes of Γ versus pump power are also observed. Validation across different pump power conditions shows agreement between the model and measurements, with an average relative deviation of 0.2169%. The multi-stage process of nuclear spin relaxation is quantified, thereby providing a robust validation for the PBR model.

## 1. Introduction

Atomic spin gyroscopes are strong candidates for the next generation navigation-grade inertial sensors [1,2]. In atomic spin gyroscopes, the spin of noble atoms is coherently driven and in situ detected by the laser beam [3,4]. According to the transition of atomic energy levels, the carrier angular rate can be detected by the frequency and phase shift in the nuclear spin. Nuclear magnetic resonance gyroscopes (NMRGs) rely on the nuclear spin coherence time of noble gases such as ^129^Xe for inertial sensing [2,5,6]. However, the operational performance of these devices is fundamentally limited by the micro-scale volume of the atomic vapor cell. The cell comprises the core sensing element and contains alkali metal, noble gas, and buffer gas [7].

Over the past decades, researchers have been dedicated to the decomposition of the total relaxation rate according to mechanisms of the Xe interaction. The wall collisions, spin–rotation coupling, magnetic field gradients, and spin exchange with the alkali atoms have been systematically identified and suppressed [8]. Bhaskar et al. pioneered the optical measurement techniques of T_2_ with the pulse magnetic field [9]. The free induction decay (FID) method was developed to evaluate T_2_ of polarized noble atoms by Hunters et al. [10]. In the study of wall collisions, the method of coating on the inner surface of the vapor cell is of great accessibility, reducing the wall spin relaxation rate [11]. Shi et al. demonstrated the optical absorption spectra relating to the nuclear spin relaxation [12]. Techniques for measuring the transverse relaxation time of Xe atoms under different optical intensities in NMRGs are proposed [13]. The transverse relaxation time (*T*_2_) is an important indicator for evaluating the characterization parameters of vapor cells. Hence, extending *T*_2_ of the noble nuclei has been a persistent research focus.

Pressure-broadening absorption spectroscopy, Faraday optical-rotation detection, and magnetic resonance linewidth analysis are established as the evaluations of NMRGs. In a Rb-Xe atomic ensemble, σ^+^-polarized light propagating perpendicular to the static magnetic field induces selective electron population of energy sublevels [14]. The pump power of the incident laser attenuates progressively with optical depth accompanied by the transfer of photon spin angular momentum to the electronic spin, which subsequently evolves through two primary mechanisms: the diffusion of spin-polarized and the spin-exchange collisions of Rb atoms [15,16]. The polarized Rb electrons generate a substantial effective magnetic field acting on the Xe nuclei, which accelerates nuclear spin relaxation and thereby degrades the signal-to-noise ratio (SNR) [17,18,19]. Furthermore, when introducing the PBR model, it should be clarified that the corresponding experimental regularity had already been observed in our coworkers’ earlier studies [20,21], although a quantitative theoretical description had not yet been established. The main contribution of the present work is therefore to provide a quantitative formulation of the pump-induced relaxation function and to experimentally verify this relationship. In this sense, the PBR model can be regarded as a quantitative extension and validation built upon previously observed experimental behavior.

Performing the classical Bloch–Torrey equations with the COMSOL Multiphysics 6.4 software, we simulated the spatial distribution of electron polarization in different pump intensities. Relaxation rates and magnetic resonance frequency shifts in the nuclear spin are measured via the FID method. A multi-stage behavior in the pumping power dependence of the ^129^Xe transverse spin relaxation rate is observed. Using the volume-averaged polarization as an intermediary variable, a pump-induced biphasic relaxation (PBR) model from linear to hyperbola is quantified.

## 2. Theoretical Framework of Pump-Induced Biphasic Relaxation (PBR)

### 2.1. Electron Spin Polarization Dynamics

In Rb-Xe NMR gyroscopes, the alkali-metal electron spin serves as the mediator between optical pumping and noble-gas nuclear polarization. The spatiotemporal evolution of the Rb electron polarization $P_{e}\left(r,t\right)$ is governed by the Bloch–Torrey equation [22]:

(1) $$\frac{dP_{e}}{dt}=\gamma_{e}P_{e}\times B+D_{e}\nabla^{2}P_{e}+R_{\mathrm{op}}\left(1-P_{e}\right)-R_{SD}P_{e}$$

where γ_e_ is the electron gyromagnetic ratio, ***B*** is the magnetic field, *D*_e_ is the diffusion coefficient of Rb atoms, *R*_op_ the optical pumping rate, and *R*_SD_ the spin-destruction rate [23].

Under steady-state conditions and weak transverse electron magnetization, the longitudinal component satisfies

(2) $$D_{e}\nabla^{2}P_{e}-(R_{\mathrm{op}}+R_{SD})P_{e}+R_{op}=0$$

where the boundary condition is

(3) $$D_{e}\nabla P_{e}=P_{e}{\left(R_{\mathrm{op}}D_{e}/2\right)}^{\frac{1}{2}}$$

Equation (2) describes a diffusion–relaxation balance and determines the spatial polarization distribution inside the vapor cell. Because the pump beam exhibits a Gaussian intensity profile and finite optical depth, $R_{\mathrm{op}}\left(r\right)$ is spatially inhomogeneous. Consequently, the steady-state electron polarization $P_{e}\left(r\right)$ is nonuniform.

The spin-destruction relaxation rate (R_SD_) originates primarily from atomic spin collisions. Its temperature dependence mainly arises from two factors. First, the alkali-metal vapor density increases exponentially with temperature, which significantly enhances the collision frequency. Second, the mean relative velocity of atoms increases with temperature, further increasing the spin-destruction collision rate. As a result, R_SD_ typically increases with temperature, and this dependence can be approximated as proportional to the product of alkali number density and the thermally averaged collision cross-section.

Neglecting the diffusion in the regime of high buffer gas, the volume-averaged electron polarization reduces to [24]

(4) $$P_{\mathrm{Rb}}=\frac{R_{\mathrm{OP}}}{R_{\mathrm{OP}}+R_{\mathrm{SD}}}$$

Since the optical pumping rate scales linearly with pump power P, the ensemble-averaged polarization becomes [25]

(5) $$P_{\mathrm{Rb}}=\frac{aP}{aP+R_{\mathrm{SD}}}$$

which is a Langmuir-type saturation function. Equation (5) provides the first key element of the PBR model: electron polarization increases linearly at low power and saturates at high power.

### 2.2. Fermi-Contact Interaction and NMR Frequency Shift

The polarized Rb electrons generate an effective magnetic field acting on Xe nuclei through the Fermi-contact hyperfine interaction. The longitudinal effective field is [26,27]

(6) $$B_{\mathrm{ef}f}=\frac{2}{3}k_{0}\mu_{0}g_{S}\mu_{B}n_{\mathrm{Rb}}\langle S_{z}\rangle$$

where *k*_0_~493 is the Rb-Xe enhancement factor [28,29], *n*_Rb_ is the Rb density, *g_s_* is the electron spin g-factor, *µ_B_* is the Bohr magneton and ⟨*S_z_*⟩ the electron spin expectation value. Since *P*_Rb_ = 2 ⟨*S_z_*⟩, the resulting ^129^Xe Larmor frequency shift is

(7) $$\Delta \omega_{\mathrm{Xe}}=\gamma_{\mathrm{Xe}}B_{eff}\propto P_{Rb}$$

where *γ*_Xe_ is the gyromagnetic ratio of ^129^Xe, and the NMR frequency shift of ^129^Xe atoms is proportional to the density of polarized atoms *n*_Rb_. This linear proportionality provides a direct and quantitative method for measuring the ensemble-averaged electron polarization.

### 2.3. Mechanism of Pump-Induced Nuclear Transverse Relaxation

The total transverse relaxation rate of ^129^Xe can be decomposed as [30]

(8) $$\Gamma =\Gamma_{0}+\Gamma_{SE}+\Gamma_{\mathrm{grad}}$$

where $\Gamma_{0}$ includes wall, van der Waals and intrinsic magnetic-gradient relaxation, $\Gamma_{SE}$ arises from binary spin-exchange collisions, and $\Gamma_{\mathrm{grad}}$ originates from polarization-induced magnetic field gradients.

(1) Spin-Exchange Contribution $\Gamma_{SE}$

When the pump power is varied, the corresponding contribution of spin-exchange ${\;\Gamma }_{SE}$ is proportional to the total density of polarized Rb atoms in the vapor cell. For the stable Rb density,

(9) $$\Gamma_{SE}\;\propto \;n_{Rb}{\bar{P}}_{\mathrm{Rb}}$$

Detailed theory can be found in Appendix A.

(2) Effective Field Gradient Contribution $\Gamma_{\mathrm{grad}}$

Optical-induced inhomogeneous polarization distribution drives coherent nuclear spin precession of Xe, which subsequently undergoes dephasing and accelerated transverse relaxation [5,31]. Due to spatially inhomogeneous electron polarization, the effective magnetic field exhibits a gradient,

(10) $$\nabla B_{eff}\;\propto \;\nabla P_{Rb}$$

because the alkali contact field is proportional to the local Rb electron polarization [32]. The effective magnetic field can be written as

(11) $$B_{\mathrm{eff}}\left(r\right)=B_{0}+b_{A}\;P_{\mathrm{Rb}}\left(r\right)\;\hat{e}$$

where *b_A_* is the contact field coefficient and $\hat{e}$ denotes the direction of the effective field. The RMS gradient of the effective field satisfies


(12) $$\begin{array}{c} G_{B}^{\mathrm{rms}}\equiv {\;\langle \;{|\;\nabla B_{\mathrm{eff}}(r)\;|}^{2}\;\rangle \;}^{1/2}={{\left|\;b_{A}\;\right|\;\langle \;|\;\nabla P_{Rb}(r)\;|}^{2}\rangle }^{1/2}. \end{array}$$

Here, ⟨⋯⟩ denotes a volume average over the vapor cell.

To explicitly introduce the mean electron polarization, we define the volume-averaged Rb polarization as

(13) $$\begin{array}{c} {\bar{P}}_{\mathrm{Rb}}\equiv \langle P_{\mathrm{Rb}}\left(r\right)\rangle =\frac{1}{V}\int_{V}P_{\mathrm{Rb}}\left(r\right)\;dV, \end{array}$$

where V is the cell volume. To connect the gradient term to the mean Rb polarization, we express the spatial polarization profile in amplitude–shape form:

(14) $$\begin{array}{c} P_{\mathrm{Rb}}\left(r\right)={\bar{P}}_{\mathrm{Rb}}\;f\left(r\right),\;\;\;\;\;\;\langle f\left(r\right)\rangle =1, \end{array}$$

where ${\bar{P}}_{\mathrm{Rb}}$ represents the volume-averaged polarization and $f\left(r\right)$ is a dimensionless shape function describing the spatial distribution.

In the motional-narrowing regime, the diffusion-induced transverse relaxation rate can be written in the generic second-order form [27]:

(15) $$\begin{array}{c} \Gamma_{\mathrm{grad}}=C_{\mathrm{geom}}\;D\;\gamma_{n}^{2}\;\langle \;|\nabla B_{\mathrm{eff}}\left(r\right){|}^{2}\;\rangle , \end{array}$$

where D is the noble-gas diffusion coefficient, $\gamma_{n}$ is its gyromagnetic ratio, and $C_{\mathrm{geom}}$ is a dimensionless geometry-dependent factor determined by the cell shape, boundary conditions, and the definition of the gradient. Substituting Equations (11)–(14) into Equation (15), the gradient relaxation rate can be expressed as

(16) $$\begin{array}{c} \langle |\nabla P_{\mathrm{Rb}}\left(r\right){|}^{2}\rangle ={\bar{P}}_{\mathrm{Rb}}^{\;2}\langle |\nabla f\left(r\right){|}^{2}\rangle . \end{array}$$

Combining Equations (12), (15), and (16), we can obtain

(17) $$\Gamma_{\mathrm{grad}}=C_{\mathrm{geom}}\;D\;\gamma_{n}^{2}\;{\bar{P}}_{\mathrm{Rb}}^{\;2}\langle |\nabla f\left(r\right){|}^{2}\rangle$$

which explicitly shows that the gradient-induced relaxation rate scales quadratically with the volume-averaged electron polarization.

This formulation makes clear that we do not assume $\nabla P_{\mathrm{Rb}}\propto P_{\mathrm{Rb}}$ pointwise. Instead, we use a scaling relation for the RMS gradient amplitude under a pump power-independent profile shape $f\left(r\right)$. Deviations from strict ${\bar{P}}_{\mathrm{Rb}}^{2}$ scaling can occur if the shape $f\left(r\right)$ changes with pump power (e.g., due to strong optical depth effects or diffusion-limited pumping). The effective magnetic field gradient arising from optical polarization represents the dominant contribution, as residual gradients along the *z*-axis are sufficiently weak to be neglected [33].

The transverse nuclear spin relaxation rate of ^129^Xe can be expressed by

(18) $$\Gamma =\Gamma_{0}+\eta {\bar{P}}_{\mathrm{Rb}}+\varepsilon {\bar{P}}_{\mathrm{Rb}}^{2}.$$

where *η* is the linear coefficient and $\Gamma_{0}$ is the constant part of relaxation that is irrelevant to *P*_Rb_. The electron polarization is

(19) $${\bar{P}}_{\mathrm{Rb}}=\frac{R_{\mathrm{OP}}}{R_{\mathrm{OP}}+R_{\mathrm{SD}}}$$

where *R*_OP_ is the optical pumping rate of the laser beam. The spin-destruction relaxation rate *R*_SD_ originates from Rb–Rb, Rb–buffer-gas (N_2_) and Rb–Xe collisions. In rate-constant form, R_SD_ = $k_{\mathrm{SD}}^{\mathrm{Rb}-\mathrm{Rb}}$·n_Rb_ + $k_{\mathrm{SD}}^{\mathrm{Rb}-N2}$·n_N2_ + $k_{\mathrm{SD}}^{\mathrm{Rb}-Xe}$·n_Xe_. The dominant temperature dependence arises from the exponential increase in Rb vapor density n_Rb_ governed by the gas pressure. Consequently, R_SD_ increases with temperature, shifting the transition boundary between linear and hyperbolic regimes in the PBR model [22]. As it stands, Γ can be transformed into

(20) $$\Gamma =\Gamma_{0}+\eta \frac{R_{\mathrm{OP}}}{R_{\mathrm{OP}}+R_{\mathrm{SD}}}+\varepsilon {\bar{P}}_{\mathrm{Rb}}^{\;2}$$

Here, *R*_SD_ is rendered as a constant when changing the pump power.

Since the optical pumping rate *R*_OP_ scales linearly with the pump power *P* [19],

(21) $$R_{\mathrm{op}}=\frac{\sigma \left(\Delta \right)P}{\hbar \omega A_{\mathrm{beam}}}$$

Here, σ(Δ) is the effective absorption cross-section and ω is the laser frequency, while *A*_beam_ is the beam area.

#### 2.3.1. Under the Condition of $R_{\mathrm{OP}}\ll R_{\mathrm{SD}}$

The electron polarization in Equation (9) can be rewritten as

(22) $$\frac{R_{\mathrm{op}}}{R_{\mathrm{op}}+R_{SD}}\approx \frac{R_{\mathrm{op}}}{R_{SD}}$$

Neglecting the second-order component which is negligibly small compared to the others, the relaxation rate exhibits a linear dependence:

(23) $$\Gamma =\Gamma_{0}+aP$$

Then, the total transverse relaxation rate Γ linearly responds to the low power at an initial slope:

(24) $$\underset{P\to 0}{\lim}\frac{d\Gamma }{dP}=a=\frac{\eta }{R_{\mathrm{SD}}}\frac{\sigma \left(\Lambda \right)}{\hbar \omega A_{\mathrm{beam}}}$$

where *a* is the pump efficiency coefficient characterizing the coupling strength between the optical pumping rate and the induced relaxation. *R*_SD_ quantifies electronic relaxation, *η* denotes the coefficient coupling electron polarization to nuclear relaxation, and the remaining quantities are determined by pump beam quality.

#### 2.3.2. Under the Condition of $R_{\mathrm{op}}\approx R_{\mathrm{SD}}$

With the elevated pump intensities, the pumping rate *R*_op_ increases and becomes comparable to *R*_SD_. The transverse relaxation rate Γ exhibits a saturation-type dependence on the pump power *P*, which can be described by the following Langmuir-type function:

(25) $$\Gamma =\Gamma_{0}+\frac{aP}{1+aP}+{\varepsilon \bar{P}}_{\mathrm{Rb}}^{\;2}$$

In the experimental testing range (P ∈ [0.1, 5] mW, *P*_Rb_ ∈ [0.05, 0.9] and *a* ∈ [0.01, 0.5] mW^−1^); ${\bar{P}}_{\mathrm{Rb}}^{\;2}$ is relatively small in magnitude and exhibits weak variation, rendering it negligible compared to other terms. When the product *aP* remains below unity, the system is in a sub-saturation regime, and the relaxation rate increases monotonically with the pump power. The slope diminishes from the initial value *a* to a concave-down saturation curve with curvature:

(26) $$\frac{d^{2}\Gamma }{dP^{2}}=-\frac{2a^{2}}{{\left(1+aP\right)}^{3}}<0$$

Expanding Equation (16) will yield an intuitive interpretation of the hyperbola relationship:

(27) $$\Gamma =\Gamma_{0}+aP-\frac{a^{2}P^{2}}{1+aP}$$

Corresponding Equation (14) as an asymptotic extension, the relaxation rate exhibits a downward deviation from this baseline that becomes increasingly pronounced as *P* increases.

#### 2.3.3. Under the Condition of $R_{\mathrm{OP}}\gg R_{\mathrm{SD}}$

As the pump power increases (the high-power limit), the transverse relaxation rate asymptotically approaches a constant saturation value. The saturation behavior indicates that beyond a critical pump power (P≫1/a), further increasing of the optical pumping will no longer significantly enhance the relaxation rate.

Physically, this saturation arises from the finite polarization capacity of the atomic ensemble. Once the electron polarization approaches unity, additional pump power cannot further increase the effective magnetic field gradients as well as the relaxation rate, leading to the observed power-independent ceiling in the high-power limit. The high-power regime is also incorporated into the PBR model as expressed in Equations (18)–(27). The threshold magnitude is related to the dynamic balance between the intrinsic relaxation processes and the atomic multiphysics coupling. Thus, the nuclear spin relaxation rate displays a multi-regime evolution as the pumping rate varies.

## 3. Materials and Methods

### 3.1. Experimental Setup

**Figure 1:**
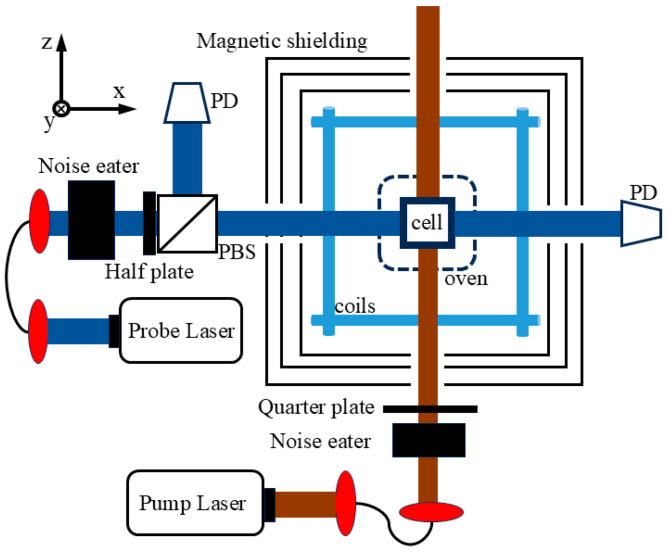
Schematic of the NMRG system.

The apparatus of NMRG is shown in Figure 1. A microfabricated glass vapor cell contains enriched-^87^Rb, 2 Torr of ^129^Xe, 8 Torr of ^131^Xe, and 280 Torr of N_2_ as the buffer gas. At the temperature of 393 K, the vapor cell is encircled by a set of magnetic field coils and a three-layer magnetic shield. Two distributed bragger reflector (DBR) lasers are used as the light source for pumping and probing. The pump laser is tuned to the Rb D_1_ line transition with a power of 2 mW, propagating along the longitudinal *z*-axis. The noise eaters are utilized to adjust and stabilize the pump and probe power, and a quarter-wave plate (QWP) is used to transform the linearly polarized light into the circularly polarized pump light. Considering the vapor cell is of 3 mm inner length, the beam diameter is set to 2.5 mm [30,31]. The probe laser is tuned to the Rb D_2_ line with the probe power of 1 mW. A balanced photodetector (Thorlabs PDB210A, Thorlabs Inc., Newton, NJ, USA ) collects the optical signals, and the Faraday rotation detection scheme is employed. Finally, the signal is demodulated by a lock-in amplifier (Zurich Instruments HF2LI, Zurich, Switzerland) to extract the frequency of atomic spin precession.

### 3.2. Measurement and Data Processing

**Figure 2:**
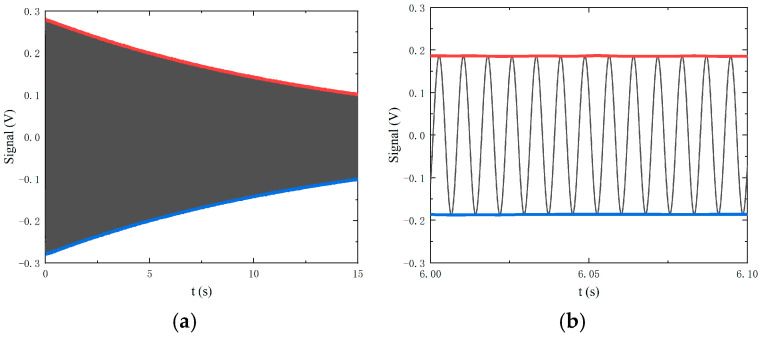
(**a**) The FID signal of ^129^Xe spin moment; (**b**) the magnified view of signals.

The transverse relaxation time *T*_2_ characterizes nuclear spin coherence; longer *T*_2_ indicates superior operational stability in NMRGs. For ^129^Xe nuclear spins, the parameter is typically measured by the FID method [17,18,19]. The nuclear spin is hyperpolarized through collisions with optically pumped Rb atoms, causing the nuclear spin to precess around the static magnetic field B_0_. A transverse π/2 pulse magnetic field is applied, and the magnetization of the nuclear spin is flipped to the transverse plane. Then, the probe laser detects the precession signal of Xe nuclear spin. The FID amplitude is recorded, and the lines are fitted as red and blue curves in Figure 2a. Revealing sinusoidal oscillations at the Larmor frequency with an exponentially decaying envelope, Figure 2b presents a magnified view of the FID signal. The transverse relaxation time *T*_2_ was extracted by fitting the FID amplitude:

(28) $$y\left(t\right)=e^{-\frac{t}{T2}}+a_{1}$$

where the equation is an exponential decay curve, and *a*_1_ is a constant value.

For each optical power and temperature condition, three independent FID measurements are performed, and the reported values correspond to the averaged results.

### 3.3. Numerical Simulation

Finite element simulations were performed using COMSOL Multiphysics 6.4 to model the three-dimensional spatial distribution of spin polarization. The pump beam irradiance is modeled as a Gaussian distribution. Simulations were conducted with a fixed beam diameter of 2 mm and the pump power ranging from 0.1 mW to 4 mW. All simulation parameters, including diffusion coefficients, absorption cross-sections, and relaxation constants, are explicitly listed in Appendix B to ensure full reproducibility of the numerical model. Diffusion coefficients are treated as effective values under the specified buffer-gas pressure and temperature conditions. Other model parameters follow those reported in our previous work [14].

These parameters are used in simulations to model the interaction of the pump beam and spin polarization with identical geometrical cell parameters to the experiments. Simulations are conducted under approximately fifteen different optical power conditions corresponding to those employed in the experiments to enable direct comparison.

## 4. Results and Discussion

**Figure 3:**
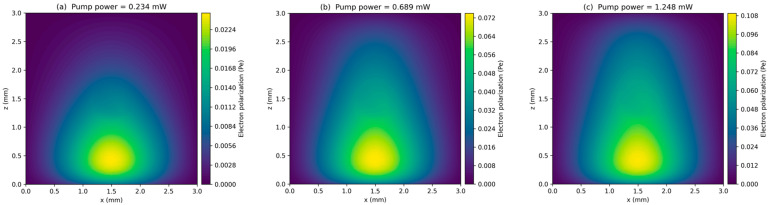
The electron polarization distribution with the pump powers of (**a**) 0.234 mW, (**b**) 0.689 mW, (**c**) 1.248 mW.

Electron polarization is primarily obtained from COMSOL simulations. For each pump power, a Bloch–Torrey-type formulation including diffusion and pumping/relaxation processes is solved, yielding a steady-state electron polarization field $P\left(r\right)$. The exported COMSOL results provide 3D point clouds (x,y,z,P). Part of the simulations are shown in Figure 3 for visualization. We compute an effective volume-averaged electron polarization using a node-averaged approximation,

(29) $$\bar{P}\left(P\right)\approx \frac{1}{N}\overset{N}{\underset{i=1}{\sum }}P(r_{i}),\;\;$$

with N=10,082 nodes per file.

**Figure 4:**
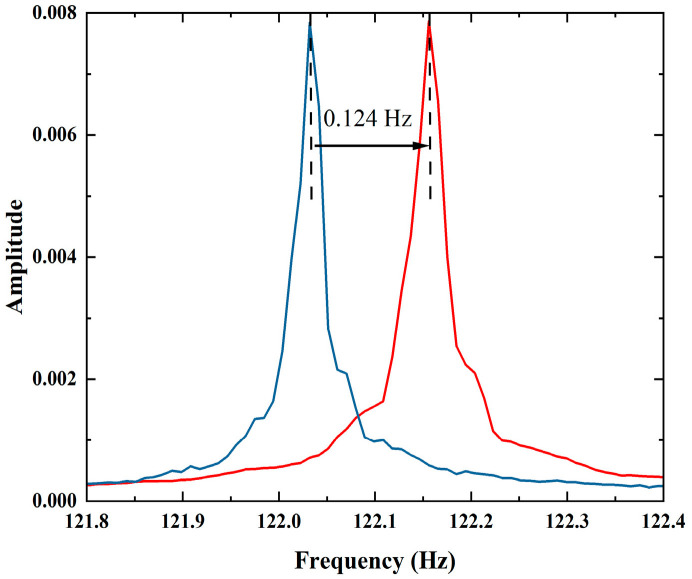
Experimental results of NMR frequency shifts. The blue line represents the FFT spectrum after a 90° flip of the linear polarization direction relative to the red line, with all other experimental conditions unchanged.

The electron polarization can be measured experimentally via xenon NMR frequency shifts arising from the Fermi-contact interaction, where the xenon frequency shift is proportional to the electron polarization. Using the apparatus illustrated in Figure 1 and performing the Fast Fourier Transform (FFT) method, the amplitude of the FID signal is plotted as a function of frequency. The main peak at the magnetic resonance frequency is measured and compared with that of the opposite electron spin direction. As shown in Figure 4, according to the relation ω = γ∙B, the bias field B is about 10 µT, corresponding to a ^129^Xe Larmor frequency of approximately 122.1 Hz. The measured frequency splitting Δω = 0.124 Hz corresponds to an effective magnetic field variation ΔB ≈ 10.5 nT. This ΔB is then converted into the averaged Rb electron polarization using Equations (6) and (7), resulting in a polarization of 13.60%.

**Figure 5:**
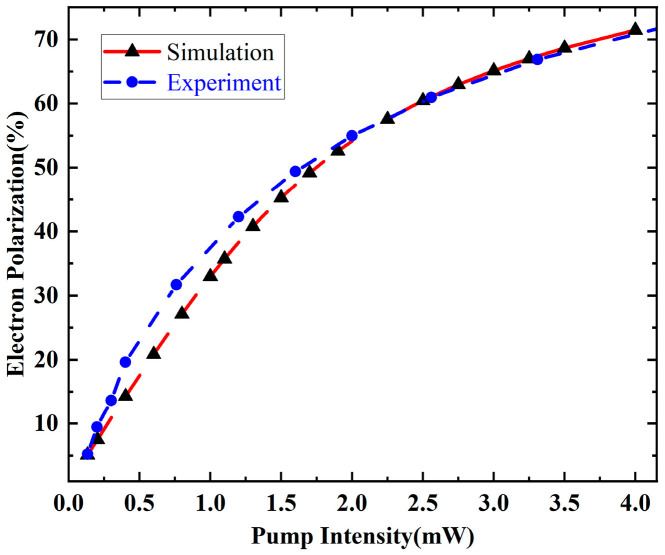
Simulation and experimental results of electron polarization under different pump intensities.

Figure 5 shows the normalized electron spin polarization as a function of the pump power that the dashed lines are drawn as a guide to the eye. It can be seen that the electron polarization rate increases with higher pump intensities while the slope diminishes rapidly towards saturation. The simulated results agree well with the experimental results. Both relations imply a Langmuir-type saturation of the volume-averaged polarization in Equation (5) which is coherent with former research [20].

This hyperbolic saturation provides the microscopic basis for the concave-down $\Gamma \left(P_{\mathrm{pump}}\right)$ behavior in the intermediate power regime (negative curvature) once Γ is expressed as a function of a saturating polarization. As the optical power increases, the electron spin polarization experiences a spatial redistribution, with the average polarization showing a monotonic increase. The simulation results suggest that the polarization’s homogeneity improves with higher light power, demonstrating the key influence of optical power in the Rb-Xe system. These simulations were compared with the experimental results, showing good agreement and highlighting the optimized parameters for better spin polarization homogeneity in the NMRG system.

**Figure 6:**
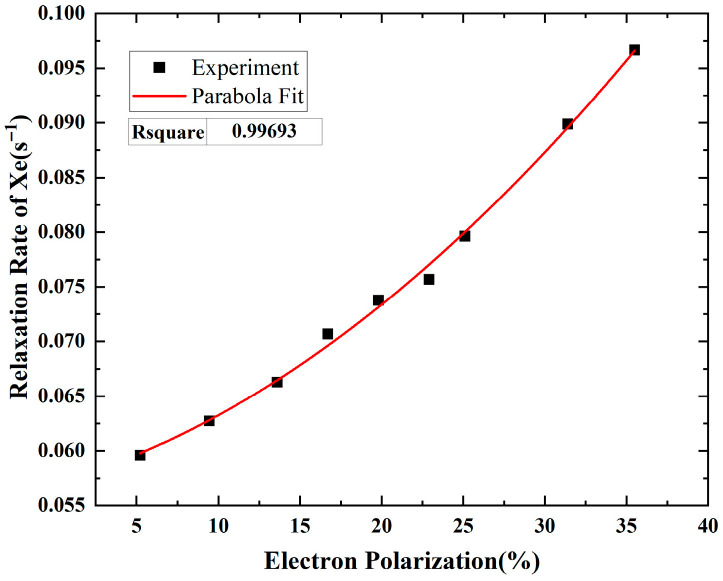
Quadric fitting to the relaxation rate of Xe spin via electron polarization.

The electron polarization is the crucial factor contributing to the ^129^Xe transverse relaxation rate at various optical pumping intensities. We employ the measured electron polarization magnitude *P*_Rb_ as an intermediate variable connecting the relationship of Γ-*P*. Consequently, experimental investigations of the Γ-*P*_Rb_ are carried out in the vapor cell as shown in Figure 6. The quadric relationship is fitted between the measured nuclear relaxation rate and electron polarization. The coefficient of determination (*R*^2^) equals 0.9969, which is consistent with the theoretical Equation (9).

In our coworkers’ previous studies, the relationship between the Rb polarization and the Xe relaxation rate was experimentally investigated in vapor cells with different parameter configurations [20,21]. The experimental data reported in those works also support the conclusion obtained in the present study. Specifically, parabolic fitting of the data yields coefficients of determination (R^2^) exceeding 0.996, indicating a highly consistent quadratic dependence between Rb polarization and the Xe relaxation rate. The detailed experimental parameters and measurement conditions can be referred to in the two studies. The experimental results verify the relationship between the relaxation rate and electron polarization, which is the foundation of the PBR model. Thus, the model can be applied to explain the relaxation rate against the pump intensity.

Further, we fit $\bar{P}\left(P_{\mathrm{pump}}\right)$ from COMSOL point clouds. $\Gamma_{129}$ and $\bar{P}$ are fitted with experimental points corresponding to the same simulation settings of optical intensities. Substituting the polarization–intensity mapping into the quadratic relaxation–polarization model yields the mechanistic, closed-form prediction $\;\Gamma_{129}\left(P_{\mathrm{pump}}\right)$. The parameters and equations are listed in Table 1 (values in parentheses are 1σ standard errors) and the fit quality is evaluated by $R^{2}$ with RMSE (Root Mean Square Error).

**Figure 7:**
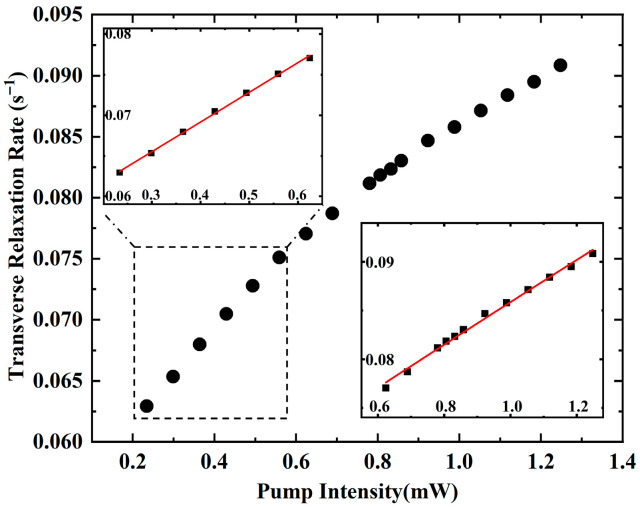
Piecewise fitting to the measured nuclear relaxation rate and pump intensity under 1.3mW. The red fitting lines in left and right insets are based on Equation (23) and Equation (27), respectively.

As is shown in Figure 7, we derived the following parameters by fitting the experimental data to the theoretical model:

α=0.0552,n=0.3835, β=0.0308

These fitted parameters accurately describe the relationship between optical intensity P and transverse relaxation rate Γ. For each data point, the relative error δ between the measured relaxation rate $\Gamma_{\exp}$ and the theoretical relaxation rate $\Gamma_{\mathrm{theo}}$ is calculated as

(30) $$\delta =\frac{|\Gamma_{\exp}-\Gamma_{\mathrm{theo}}|}{\Gamma_{\exp}}\times 100\%$$

Table 2 summarizes the results.

**Figure 8:**
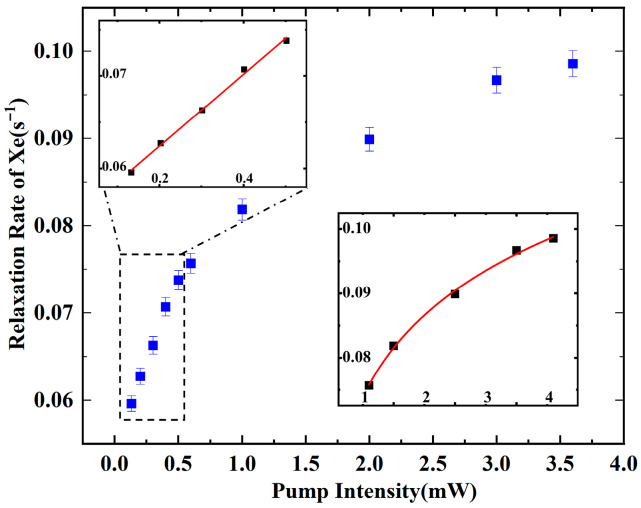
Piecewise fitting to the measured nuclear relaxation rate and pump intensity (0.2–4 mW). Error bars indicate data distribution of three repeated measurements, and blue dots represent the average values.

To ensure experimental reproducibility, we validated the PBR model using a wider power range while maintaining identical experimental conditions, as shown in Figure 8. When the optical intensity is over 3.6 mW, the ^129^Xe transverse relaxation rate is enlarged with a minor magnitude. Moreover, when the optical intensity is below 1 mW, the transverse relaxation rate increases linearly with the corresponding pump beam intensity. In summary, the relaxation rate of the ^129^Xe nuclear spin first grows linearly and then gradually increases, approaching saturation with a diminishing slope. Across the different pumping intensities, the transverse relaxation rate evolves from a linear to a hyperbola dependence on the pump intensity. As shown in the inset of Figure 8, the linear regression is performed in the low-intensity regime, and the fitted result (*R*^2^ = 0.9966) exhibits satisfactory agreement with the PBR model.

We refer to the combined model as the Arrhenius–PBR (APBR) global model as

(31) $$\Gamma \left(P,T\right)=\Gamma_{0}\left(T\right)+\eta \frac{P}{P+P_{0}\left(T\right)}+\varepsilon \left(T\right){\left(\frac{P}{P+P_{0}\left(T\right)}\right)}^{2}$$

Here, $P_{0}\left(T\right)=R_{SD}/\alpha$ represents the transition scale corresponding to $R_{OP}∼R_{SD}$, i.e., the pump power threshold where the saturation-like behavior emerges. Temperature effects are summarized by an Arrhenius law for $P_{0}\left(T\right)$,

(32) $$P_{0}\left(T\right)=P_{0,\infty }e^{-\frac{E_{a}}{RT}}$$

where R is the gas constant and $E_{a}$ is the apparent activation energy associated with the thermally activated relaxation/transition process captured by $P_{0}\left(T\right)$.

(33) $$\Gamma \left(P,T\right)=\Gamma_{0}\left(T\right)+\eta \frac{P}{P+P_{0,\infty }e^{-\frac{E_{a}}{RT}}}+\varepsilon \left(T\right){\left(\frac{P}{P+P_{0,\infty }e^{-\frac{E_{a}}{RT}}}\right)}^{2}$$

Here, Γ_0_(T) and ε(T) are temperature-related parameters, while η is shared globally.

Theoretical predictions and experimental observations are found to be in good agreement, indicating that the proposed piecewise fitting scheme aligns with the PBR model, characterized by an evolution from linear to quadratic behavior. Upon further increase in pump intensity, both the electron polarization and the nuclear spin relaxation rate approach their respective saturation values.

## 5. Conclusions

The PBR model describing the multi-stage pump-induced spin relaxation of ^129^Xe nuclear spin is proposed, with the linear to hyperbolic transition quantified in NMRGs. Performing the classical Bloch–Torrey equations with the COMSOL Multiphysics software, the spatial distribution of electron polarization in different pump intensities is illustrated. The volume-averaged electron polarization *P*_Rb_ is utilized as an intermediate variable and the transverse spin relaxation rate Γ of ^129^Xe nuclear spin is measured via the FID method. The results demonstrate that the theoretically predicted quadratic relationship between Γ and *P*_Rb_ is experimentally verified, achieving coefficients of determination (*R*^2^) of 0.9969. Linear (*R*^2^ > 0.9966) and subsequent hyperbolic (*R*^2^ > 0.9942) relationships of Γ and *P* are verified. The reliability of the PBR model is subsequently validated and the average relative error between experiment and theory is 0.2169%. The multi-stage process of nuclear spin relaxation is quantified, thereby providing a robust validation for the PBR model. This study builds upon previous work by providing quantification and validation. Further investigation will provide theoretical foundations for investigating nuclear spin relaxation mechanisms induced by optical pumping in NMRGs.

## Figures and Tables

**Table 1:** The fitting and relative deviation of experiments and the PBR theory.

Model	Equation	Parameters	R^2^	RMSE $\left(s^{-1}\right)$
polarization–intensity	$\bar{P}=P_{\mathrm{sat}}\frac{P_{\mathrm{pump}}}{P_{\mathrm{pump}}+P_{1/2}}$	$P_{\mathrm{sat}}=0.21348\pm 0.01438\;\mathrm{mW}$	0.9932	$1.56\times 10^{-3}$
		$P_{1/2}=1.82930\pm 0.18041$ mW		
relaxation–polarization	$\Gamma_{129}=\Gamma_{0}+\eta \;\bar{P}+\xi \;{\bar{P}}^{2}$	$\Gamma_{0}=0.05588\pm 0.00137s^{-1}$	0.9926	$6.91\times 10^{-4}$
		$\eta =0.31353\pm 0.05267s^{-1}$		
		$\xi =1.13023\pm 0.47654s^{-1}$		
relaxation–intensity	$\Gamma_{129}=\Gamma_{0}+a\cdot P_{\mathrm{pump}}+\frac{a^{2}\cdot P_{pump}^{2}}{1+a\cdot P_{\mathrm{pump}}}$	$\Gamma_{0}=0.6361\pm 0.00168s^{-1}$	0.9942	$5.72\times 10^{-4}$
		$a=0.02276\pm 0.023996{(s\cdot \mathrm{mW})}^{-1}$		

**Table 2:** The FID results, the PBR theoretical values and the relative error δ.

P (mW)	Γ_exp_ (s^−1^)	Γ_theo_ (s^−1^)	δ (%)
1.248	0.09085	0.09087	0.1192
1.183	0.08950	0.08981	0.2653
1.118	0.08842	0.08886	0.0617
1.053	0.08714	0.08723	0.0293
0.988	0.0858	0.08585	0.00665
0.923	0.08468	0.08498	0.3430
0.858	0.08304	0.08313	0.1560
0.832	0.08235	0.08251	0.0614
0.806	0.08186	0.08193	0.2240
0.780	0.08116	0.08123	0.1463
0.689	0.07871	0.07875	0.00189
0.624	0.07704	0.07717	0.1515
0.559	0.07511	0.07515	0.1207
0.494	0.07278	0.07298	0.2658
0.429	0.07049	0.07069	0.3746
0.364	0.06799	0.06813	0.4798
0.299	0.06536	0.06549	0.3557
0.234	0.06294	0.06306	0.7407

## Data Availability

The original contributions presented in this study are included in the article. Further inquiries can be directed to the corresponding author.

## Appendix A. Derivation Spin-Exchange Relaxation

In spin-exchange optical pumping (SEOP), the dominant spin-dependent interaction between ^87^Rb valence electron spin S and ^129^Xe nuclear spin $I_{b}$ arises from the isotropic hyperfine (Fermi-contact) coupling during a binary collision. Following the standard treatment, the spin-dependent part of the interatomic interaction is written as [34]

(A1) $$V_{1}(R)\;=\;g(R)\;N\cdot S\;+\;A_{b}(R)\;I_{b}\cdot S$$

where R is the internuclear separation along a classical collision trajectory $R\left(t\right)$, N is the rotational angular momentum of the collision complex, $g\left(R\right)$ is the spin–rotation coupling, and $A_{b}\left(R\right)$ is the isotropic hyperfine coupling coefficient responsible for spin exchange (angular momentum transfer between S and $I_{b}$).

In what follows, we focus on the isotropic term $A_{b}\left(R\right)\;I_{b}\cdot S$, since it is the term that directly drives polarization transfer between Rb electron spins and Xe nuclear spins in SEOP.

Under SEOP conditions of interest (high buffer-gas pressure and frequent collisions) [35,36,37], the spin evolution due to $V_{1}\left(R\right)$ can be treated in a binary collision, Markovian framework, and the alkali ensemble can be taken in the spin-temperature limit [38]. In this limit, Walker and Happer derive coupled rate equations for the ensemble-averaged longitudinal spin components $\left\langle S_{z}\right\rangle$ and $\left\langle I_{bz}\right\rangle$ produced by collisions under $V_{1}$ [34].

Specifically, the noble-gas nuclear spin evolves as

(A2) $$\begin{array}{c} \frac{d\langle I_{bz}\rangle }{dt}=G_{b}\left(A_{b}\right)\;\left[\varepsilon \left(I_{b},\beta_{b}\right)\;\langle S_{z}\rangle -\langle I_{bz}\rangle \right], \end{array}$$

where $G_{b}\left(A_{b}\right)$ is the spin-exchange collision rate (a characteristic rate determined by the microscopic interaction and collision statistics), and $\varepsilon \left(I_{b},\beta_{b}\right)$ is a dimensionless factor that depends on the noble-gas spin and spin-temperature parameters (details in the cited reference).

For the important case $I_{b}=\frac{1}{2}$ (e.g., ^3^He and ^129^Xe), Equation (A2) simplifies to

(A3) $$\begin{array}{c} \frac{d\langle I_{bz}\rangle }{dt}=-\;G_{b}\left(A_{b}\right)\;\left(\left\langle I_{bz}\rangle -\langle S_{z}\right\rangle \right). \end{array}$$

Equation (A3) shows that spin exchange drives the noble-gas nuclear spin toward the alkali electron spin, with a characteristic rate $G_{b}\left(A_{b}\right)$. Importantly for our purpose, the source term is proportional to $\left\langle S_{z}\right\rangle$, i.e., proportional to the alkali polarization.

The same microscopic derivation gives an explicit expression for the characteristic rate $G_{b}\left(A_{b}\right)$ as an average over binary collision parameters (impact parameter b, relative velocity w, and classical trajectory $R\left(t\right)$) [23]:

(A4) $$\begin{array}{c} G_{b}\left(A_{b}\right)=n_{a}\;\frac{\pi }{2}\int_{0}^{\infty }db\;b\int d^{3}w\;f\left(w\right)\;w^{3}|\int_{-\infty }^{+\infty }dt\frac{A_{b}R\left(t\right))}{\hbar }{|}^{2}, \end{array}$$

where $n_{a}$ is the alkali number density (in this work $n_{a}=n_{\mathrm{Rb}}$) and $f\left(w\right)$ is the Maxwell–Boltzmann distribution of relative velocities. Equation (A4) makes the dependence

(A5) $$\begin{array}{cc} G_{b}\left(A_{b}\right)\propto n_{\mathrm{Rb}} & \end{array}$$

explicit (all remaining factors depend on temperature and the microscopic potential/coupling through $A_{b}\left(R\right)$ and the collision ensemble, but not on the alkali polarization itself in the lowest-order binary collision).

It is common to package Equation (A4) into the “rate constant” form:

(A6) $$\begin{array}{c} G_{b}\left(A_{b}\right)\equiv \gamma_{\mathrm{SE}}=k_{\mathrm{SE}}\;n_{\mathrm{Rb}},k_{\mathrm{SE}}=\langle \sigma_{\mathrm{SE}}v\rangle , \end{array}$$

where $k_{\mathrm{SE}}$ is the spin-exchange rate coefficient (thermal average of cross-section times relative speed). Equation (A6) is exactly the structure used in standard SEOP rate-equation summaries, e.g., for ^3^He,

(A7) $$\begin{array}{c} \frac{dP_{\mathrm{He}}}{dt}=k_{\mathrm{SE}}\left[A\right]\;\left(P_{A}-P_{\mathrm{He}}\right)-\Gamma_{\mathrm{He}}P_{\mathrm{He}}. \end{array}$$

Define the conventional dimensionless polarizations (for $S=\frac{1}{2}$ and $I_{b}=\frac{1}{2}$):

(A8) $$\begin{array}{c} P_{\mathrm{Rb}}\equiv \frac{\left\langle S_{z}\right\rangle }{S}=2\langle S_{z}\rangle ,P_{b}\equiv \frac{\left\langle I_{bz}\right\rangle }{I_{b}}=2\langle I_{bz}\rangle . \end{array}$$

Multiply Equation (A3) by 2 and substitute Equation (A8):

(A9) $$\begin{array}{c} \frac{dP_{b}}{dt}=-\;G_{b}\left(A_{b}\right)\;\left(P_{b}-P_{\mathrm{Rb}}\right)=G_{b}\left(A_{b}\right)\;\left(P_{\mathrm{Rb}}-P_{b}\right). \end{array}$$

Using Equation (A6) ($G_{b}\left(A_{b}\right)=k_{\mathrm{SE}}n_{\mathrm{Rb}}$), we obtain the standard SEOP form:

(A10) $$\begin{array}{c} \frac{dP_{b}}{dt}=k_{\mathrm{SE}}\;n_{\mathrm{Rb}}\;\left(P_{\mathrm{Rb}}-P_{b}\right). \end{array}$$

If $P_{b}\ll P_{\mathrm{Rb}}$, then from Equation (A10)

(A11) $$\begin{array}{c} \frac{dP_{b}}{dt}{|}_{P_{b}\approx 0}\approx k_{\mathrm{SE}}\;n_{\mathrm{Rb}}\;P_{\mathrm{Rb}}. \end{array}$$

Spin-exchange relaxation (build-up) rate $\Gamma_{\mathrm{SE}}$ is the spin-exchange driven polarization change rate in the small-$P_{b}$ limit (i.e., the source term that sets the initial slope of the noble-gas polarization buildup),

(A12) $$\begin{array}{c} \Gamma_{SE}\equiv {\left|\frac{dP_{b}}{dt}\right|}_{P_{b}\approx 0}, \end{array}$$

then Equation (A11) gives the required proportionality:

(A13)
$$\Gamma_{\mathrm{SE}}\;=k_{\mathrm{SE}}\;n_{\mathrm{Rb}}\;\;P_{\mathrm{Rb}}\propto n_{\mathrm{Rb}}\;P_{\mathrm{Rb}}.$$

At fixed Rb number density $n_{\mathrm{Rb}}$, this reduces to

(A14) $$\begin{array}{c} \Gamma_{\mathrm{SE}}\propto P_{\mathrm{Rb}}. \end{array}$$

Equation (A13) states that the spin-exchange driven nuclear polarization change rate is proportional to $n_{\mathrm{Rb}}$ (more collisions per unit time), and $P_{\mathrm{Rb}}$ (more oriented electron angular momentum available to transfer per collision). This follows directly from the microscopic collision-averaged rate Equation (A3) and the density scaling of $G_{b}\left(A_{b}\right)$ in Equation (A4).

## Appendix B. The COMSOL Multiphysics Parameters Used in This Work

**Table A1:** The parameters used in the COMSOL Multiphysics 6.4 simulation.

Symbol	Description	Value	Unit
L	Vapor cell inner length	3	mm
d_beam_	Pump beam diameter	2	mm
R_e_	Total electron relaxation rate	11,840	s^−1^
R_p_	Optical pumping rate (nominal)	2.3 × 10^5^	s^−1^
σ_op_	Effective absorption cross-section	9 × 10^−17^	m^2^
n_Rb_	Rb atomic number density	2 × 10^19^	m^−3^
D_Rb_	Rb diffusion coefficient	1.2 × 10^−3^	m^2^/s
D_Xe_	Xe diffusion coefficient	2.5 × 10^−5^	m^2^/s
R_se_	Rb–Xe spin-exchange rate	0.484	s^−1^
